# Most common reasons for primary care visits in low- and middle-income countries: A systematic review

**DOI:** 10.1371/journal.pgph.0000196

**Published:** 2022-05-02

**Authors:** Jacob Bigio, Emily MacLean, Nathaly Aguilera Vasquez, Lavanya Huria, Mikashmi Kohli, Genevieve Gore, Emma Hannay, Madhukar Pai, Pierrick Adam

**Affiliations:** 1 Research Institute of the McGill University Health Centre, Montreal, Canada; 2 McGill International TB Centre, Montreal, Canada; 3 Dept of Epidemiology and Biostatistics, McGill University, Montreal, Canada; 4 Schulich Library of Physical Sciences, Life Sciences, and Engineering, McGill University, Montreal, Canada; 5 Foundation for Innovative New Diagnostics, Geneva, Switzerland; 6 Manipal Academy of Higher Education, Manipal University, Manipal, India; 7 Infectious Diseases Programs Control Unit, Ministry of Health, Tahiti, French Polynesia; University of the Witwatersrand, SOUTH AFRICA

## Abstract

With the Covid-19 pandemic and the introduction of the WHO’s Essential Diagnostics List (EDL), increasing global attention is focused on the crucial role of diagnostics in achieving universal health coverage. To create national EDLs and to aid health system planning, it is vital to understand the most common conditions with which people present at primary care health facilities. We undertook a systematic review of the most common reasons for primary care visits in low- and middle-income countries. Six databases were searched for articles published between January 2009 and December 2019, with the search updated on MEDLINE to January 2021. Data on the most common patient reasons for encounter (RFEs) and provider diagnoses were collected. 17 of 22,279 screened articles were included. Most studies used unvalidated diagnostic classification systems or presented provider diagnosis data grouped by organ system, rather than presenting specific diagnoses. No studies included data from low-income countries. Only four studies (from Brazil, India, Nigeria and South Africa) using the ICPC-2 classification system contained RFE and provider diagnosis data and could be pooled. The top five RFEs from the four studies were headache, fever, back or low back symptom, cough and pain general/multiple sites. The top five diagnoses were uncomplicated hypertension, upper respiratory tract infection, type 2 diabetes, malaria and health maintenance/prevention. No psychological symptoms were among the top 10 pooled RFEs. There was more variation in top diagnoses between studies than top RFEs, showing the importance of creating location-specific lists of essential diagnostics for primary care. Future studies should aim to sample primary care facilities from across their country of study and use ICPC-3 to report both patient RFEs and provider diagnoses.

## Introduction

Primary health care (PHC) is a major point of entry into healthcare systems for people seeking care. Defined by the World Health Organization (WHO) as a whole-of-society approach to health that focuses on people’s needs as early as possible along the continuum of health and as close as feasible to their everyday environment [1], PHC is recognised as a cornerstone of achieving universal health coverage (UHC) and meeting the health-related Sustainable Development Goals [1, 2]. According to the WHO, scaling up PHC interventions across low- and middle-income countries (LMICs) could save 60 million lives and increase average life expectancy by 3.7 years by 2030 (2).

In recent years and especially with the ongoing Covid-19 pandemic, increasing global attention has been focused on the crucial role of diagnostics in high-quality healthcare systems, including PHC, with the introduction of the WHO’s annual Essential Diagnostics List (EDL) [3] and the formation of the Lancet Commission on Diagnostics [4]. Poor access to diagnostics, particularly in LMICs, can lead to lack of trust in health services and under-utilization of services, patients being started on presumptive or empiric treatment, which can lead to poor health outcomes, waste resources and contribute to antimicrobial resistance in the case of infectious diseases [4, 5]. Lack of diagnostics is also a major concern for managing common non-communicable diseases.

The Covid-19 pandemic has further highlighted the importance of diagnostics in curbing transmission of the virus. There has been unprecedented global collaboration through mechanisms such as the Access to Covid-19 Tools (ACT)-Accelerator Diagnostics Pillar, which is co-convened by the Foundation for Innovative New Diagnostics (FIND) and the Global Fund and aims to accelerate development, equitable allocation and delivery of diagnostic tests for Covid-19 worldwide [6].

The WHO EDL acts as a policy tool for countries to create their own national EDLs based on local contexts and needs, a process so far undertaken by Bangladesh, India, Nigeria and Pakistan [7, 8]. To create such a national EDL, and to aid health system planning, resource allocation and the training of healthcare workers, it is vital to understand both the most common symptoms with which patients present to primary care, often known as patient reasons for encounter (RFEs), and the most common provider diagnoses. Along with other sources of data such as the major causes of death and disability in a country, this information will guide the range of diagnostics required at the primary care level. Knowing the most common reasons for primary care will allow WHO, FIND and country governments to develop a package of essential tests for primary care.

The WHO is currently in discussions with other LMICs, mostly in Africa, to create their own national EDLs [7]. However, little published information is available on the reasons for primary care visits in LMICs. To our knowledge, only one systematic review, published in 2018, has summarized data on reasons for primary care visits globally [9]. It included data from only three LMICs (India, Serbia and South Africa) and pooled all studies together so the most common reasons for PHC visits in LMICs could not be distinguished from the global data which mostly focused on high-income countries (HICs). Our systematic review provides an updated summary of the reasons for primary care visits, focusing exclusively on LMICs.

## Methods

The protocol for this review was registered at the International Prospective Register of Systematic Reviews (PROSPERO), identifier CRD42020159469 [10]. Patients or the public were not involved in the design, or conduct, or reporting, or dissemination plans of our research.

### Search strategy

In our initial searches, MEDLINE, EMBASE, Global Health, Web of Science Core Collection, CINAHL, LILACS were searched for papers published between 1^st^ January, 2009 and 12^th^ December, 2019. Subsequently, we updated the search on MEDLINE until 1^st^ January 2021. The search strategy was developed in consultation with a librarian (GG) and based on terms relating to “primary care” and “conditions” or “reasons” for the visit [S1 Appendix]. No restrictions on language were applied to the search. We also reviewed the papers included in the previous systematic review on this topic [9].

### Study selection

Four reviewers (JB, NAV, LH and PA) conducted the title/abstract screening, with each title/abstract independently screened by a combination of two of the four reviewers. Full text screening of included titles/abstracts was independently conducted by two reviewers (JB and PA). Articles were assessed using pre-defined inclusion and exclusion criteria, with conflicts resolved through discussion between reviewers.

Quantitative observational studies and mixed-methods studies with a quantitative observational component were included. Qualitative studies, modelling studies, economic evaluations, interventional studies and case-control studies were excluded. Studies conducted in primary care settings in LMICs (defined using the World Bank classification system [11]) reporting a minimum of five distinct RFEs or provider diagnoses were included. Studies with a data collection period of at least three months were included, though data collection did not have to occur continuously throughout the three months. Studies focusing only on specific types of visits (e.g. follow-up visits for acute conditions, referred visits, routine examinations) were excluded. Studies that selected the population based on particular symptoms (e.g. patients with fever, children with respiratory symptoms) were excluded. Studies that selected the population based on particular morbidities (e.g. HIV-positive patients, diabetic patients) or were conducted exclusively in specialized care settings (e.g. sexually transmitted infection clinics, specialized medical departments) were excluded. Studies in which data collection occurred before 1^st^ January, 2009 were excluded. Studies not published in English or French were excluded. Editorials, commentaries, conference abstracts and grey literature were excluded.

### Data extraction

Data were extracted using a standardised extraction form in Google Forms, which was piloted beforehand. Three reviewers (JB, EM and PA) conducted the data extraction, with the data from each paper independently extracted by a combination of two of the three reviewers. Extracted data were compared and any discrepancies were resolved through discussion between the reviewers. Extracted data included: country of study, data collection period, type of healthcare facility, type of healthcare providers, healthcare sector, patient demographics, source of outcome data, classification system used, total number of visits or patients, number of conditions reported, frequency of different conditions.

### Quality assessment

A quality assessment tool was adapted from the work of Hoy et al [12] for use in this study. The tool has seven domains, assessing: whether the study’s target population was a close representation of the national population; whether the PHC facilities sampled were a close representation of the PHC facilities in the target area; how the sample of patient visits was chosen; how the sample of healthcare workers was chosen; whether the same mode of data collection was used for all subjects; whether the outcome measures were consistently and validly recorded; and whether the numerators and denominators were appropriate. [S2 Appendix].

Quality assessment was conducted independently by two reviewers (JB and PA) for all included studies. Disagreements were resolved through discussion between the reviewers. All assessed studies were included, regardless of the quality assessment results.

### Data analysis

Different studies used different disease classification systems, including the International Classification of Primary Care version 2 (ICPC-2), the International Classification of Diseases 10th revision (ICD-10) and in-house classification systems. ICD-10 classifies diseases by provider diagnosis, such as essential (primary) hypertension (I10). ICPC-2 classifies diseases by patient RFE, such as fever (A03), and by provider diagnosis, such as hypertension, uncomplicated (K86).

Both ICD-10 and ICPC-2 are organised into chapters based on body systems, such as Chapter IX: Diseases of the circulatory system, and some studies presented summed totals of the number of provider diagnoses in each chapter. Such summed totals by chapter were not considered useful for the purposes of this review, as they do not give sufficient detail about the precise provider diagnoses or RFEs to help inform health system planning or the creation of national EDLs, and so they were not pooled.

Studies were pooled if at least three studies using the same classification system presented data on diagnoses or RFEs. Data from studies using different classification systems were not pooled, as it is not possible to reliably convert between different classification systems without access to individual patient data. Studies using in-house classification systems were not pooled.

Data were pooled via a rank sum system. For each study, provider diagnoses and patient RFEs were ranked from most to least common. The number of provider diagnoses ranked was determined by the study that reported the lowest number of provider diagnoses. For example, of the studies that used ICPC-2, the lowest number of provider diagnoses reported was 10 so the top 10 provider diagnoses in each study were ranked. The most common provider diagnosis in each study was assigned rank 10, the second most common chapter was assigned rank 9 and so on. Rankings from each study were combined and mean ranks were determined. The same process was followed for ranking RFEs.

## Results

### Study selection

**Fig 1:**
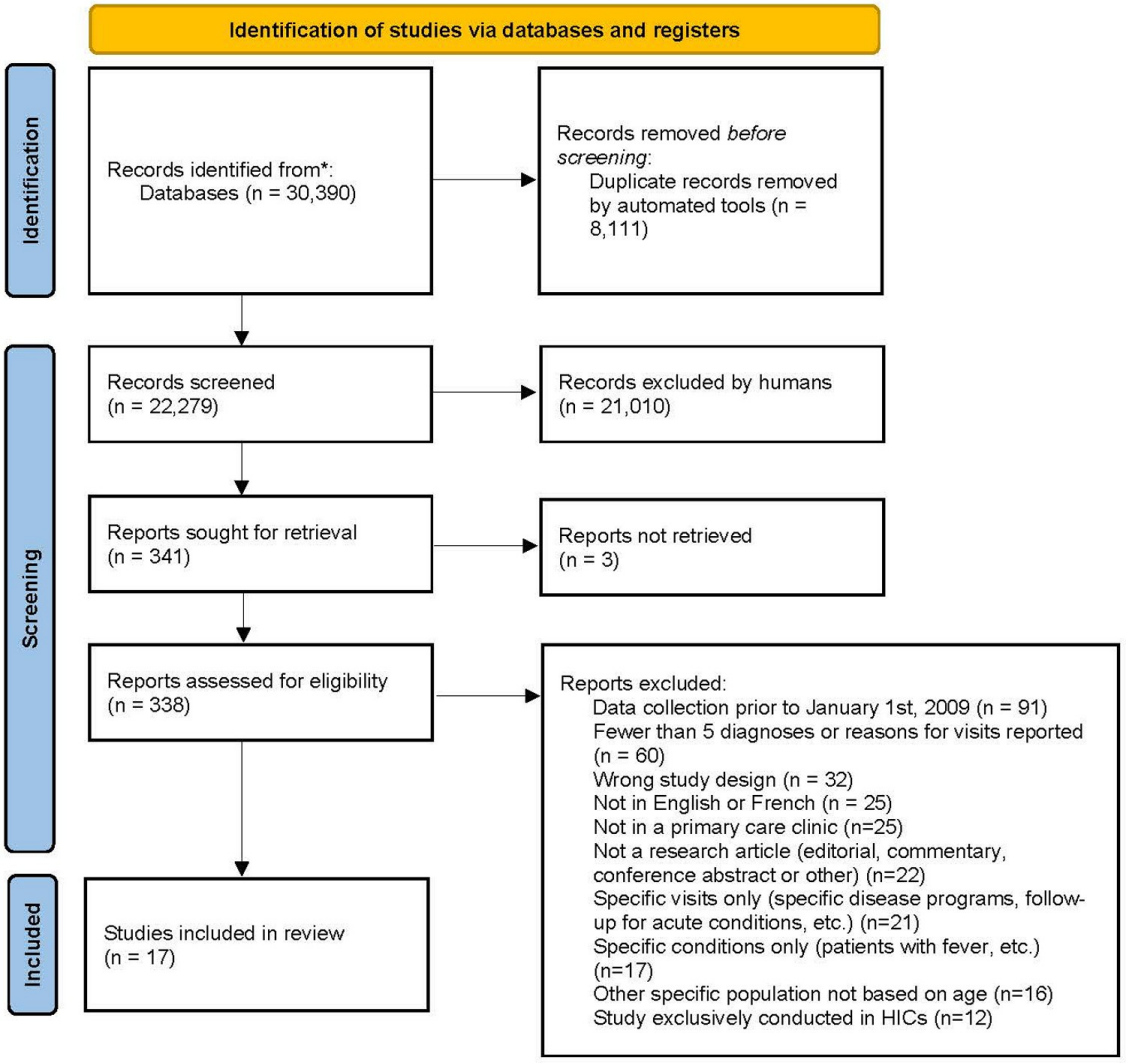
PRISMA flow diagram.

After deduplication, 22,279 records were identified. A total of 21,938 records were excluded after title and abstract screening. Of the remaining 341 studies, three full-texts could not be retrieved and a further 321 were excluded after full-text review. The top three reasons for full-text exclusion were data collection that took place before January 1^st^, 2009, studies reporting fewer than five diagnoses or reasons for visits and studies being of the wrong design (e.g. case-control studies) [Fig 1]. 17 studies were included in the review. Detailed study characteristics are shown in Table 1.

**Table 1:** Characteristics of included studies.

Study	Country	Healthcare facilities	Year	Sampling duration	Total number of visits, diagnoses included	Types of visits included	Healthcare providers	Diagnostic classification system	Data source	Age range of patients in years
Begum 2017 [13]	Bangladesh	1 facility in 1 rural area	2014–2015	Purposive selection over a period of 24 months	310 visits, 310 diagnoses	Unclear	Unclear	In-house (diagnoses by chapter)	Provider questionnaire	0–19
Chueiri 2020 [14]	Brazil	Unclear facilities in all 5 geographic regions	2016	6 months	6,160 visits, 8046 RFEs	All	Physicians	ICPC-2 (specific diagnoses and patient RFEs)	Patient interviews	Age 18+
Doyle 2019 [15]	South Africa	3 public facilities and 1 mobile facility in 1 rural district	2015–2016	6 separate months of a 12-month period	Unclear visits, 3,437 diagnoses	All	Unclear	In-house (diagnoses by chapter)	Medical records	10–24
Enato 2012 [16]	Nigeria	2 public facilities in 1 rural local government area of 1 state	2009	6 continuous months	495 visits, 512 diagnoses	All	CHWs, nurses, physicians, midwives, pharmacists	Unclear (diagnoses by chapter and specific diagnoses)	Medical records	Not reported
Gupta 2014 [17]	India	1 facility in 1 union territory	2011	12 months	Unclear visits, 68,818 diagnoses	First visits only	Physicians	ICD-10 (diagnoses by chapter)	Medical records	All ages
Gupta 2015 [18]	India	1 public facility in 1 urban area	2014	12 continuous months	Unclear visits, 6,685 diagnoses	First only	Physicians	ICD-10 (diagnoses by chapter)	Medical records	All ages
Kamarudin 2012 [19]	Malaysia	3 public facilities in 1 state	2010	12 continuous months	73,236 visits, 107,016 diagnoses	All	Physicians	ICD-10 (specific diagnoses)	Medical records	All ages
Kshirsagar 2019 [20]	India	1 facility in 1 rural area	2018	12 continuous months	13,279 visits, 12,279 diagnoses	All	Unclear	In-house (diagnoses by chapter)	Provider questionnaire	All ages
Kumar 2018 [21]	India	1 public facility in 1 urban area	2016	12 continuous months	Unclear visits, 16,483 diagnoses	First only	Unclear	In-house (diagnoses by chapter)	Medical records	All ages
Mash 2012 [22]	South Africa	112 public facilities, both rural and urban, in 4 provinces	Unclear	5 separate days per facility spread over 12 months	18,856 visits, 31,451 diagnoses	All	Nurses, physicians	ICPC-2 (diagnoses by chapter and specific diagnoses, patient RFEs)	Provider questionnaire	0–79
Merali 2014 [23]	Cambodia	1 NGO-run mobile facility visiting 19 rural villages in 5 provinces	2008–2012	50 continuous months	30,882 visits, unclear diagnoses	All	Nurses, physicians, midwives	In-house (specific diagnoses)	Medical records	All ages
Mohan 2014 [24]	India	1 public facility in 1 rural district	2013	12 continuous months	Unclear visits, 39,321 diagnoses	Unclear	Unclear	In-house (specific diagnoses) / ICD-10 (diagnoses by chapter)	Unclear	All ages
Olagundoye 2016 [25]	Nigeria	1 public facility in 1 urban area	Unclear	3 continuous months	401 visits, 546 diagnoses	All	Physicians	ICPC-2 (specific diagnoses and patient RFEs)	Medical records	Unclear
Prabhune 2017 [26]	India	1 NGO-run facility in 1 rural district	2011–2016	60 continuous months	16,487 visits, unclear diagnoses	All	CHWs, AYUSH doctors	Other validated classification (diagnoses by chapter)	Medical records	All ages
Silva 2014 [27]	Brazil	204 public family health strategy facilities in 1 macroregion	2011	1 day per team over 6 months	4,192 visits, 4,099 diagnoses/RFEs	All	Nurses, physicians	ICPC-2 (diagnoses by chapter)	Provider questionnaire	Age 11+
Swain 2017 [28]	India	4 public facilities in 1 urban area	2014	7 weeks randomly spread over 12 months	2,249 visits, 2,023 diagnoses, 2,603 RFEs	First only	Nurses, physicians	ICPC-2 (specific diagnoses and patient RFEs)	Medical records	Age 18+
Torres 2015 [29]	Brazil	4 public facilities in 1 urban area	2010–2011, 2012–2013	6 continuous months in 2010–11, 9 continuous months in 2012–13	478 visits, 478 diagnoses, 628 RFEs	All	Physicians	ICPC-2 (diagnoses by chapter) / In-house (patient RFEs)	Provider questionnaire	Age 18+

Diagnoses by chapter = summed total of number of provider diagnoses by diagnostic classification system chapter or body system is presented; specific diagnoses = specific conditions diagnosed by the healthcare provider are presented; specific RFEs = specific reasons for encounter of patients are presented; CHW = community health worker; AYUSH = Ayurveda, Yoga, Naturopathy, Unani, Siddha, Sowa-Rigpa and Homoeopathy; ICPC-2 = International Classification of Primary Care, 2nd edition; ICD-10 = International Classification of Diseases, 10th revision.

Included studies represented seven LMICs: Bangladesh (n = 1), Brazil (n = 3), Cambodia (n = 1), India (n = 7), Malaysia (n = 1), Nigeria (n = 2) and South Africa (n = 2). Each of these countries is a middle-income country (MIC)—no studies had data from low-income countries (LICs).

The number of healthcare facilities per study ranged from one to 204 (median one, interquartile range (IQR) 1–4). The number of visits reported in each study ranged from 310 to 73,236 (median 3,294, IQR 487–17,672) and was not reported for five studies. The number of diagnoses reported in each study ranged from 310 to 107,016 (median 5,692, IQR 546–31,451) and was not reported for two studies. The sources of data were medical records for 10 studies [15–19, 21, 23, 25, 26, 28], provider questionnaire for five studies [13, 20, 22, 27, 29], patient interview for one study [14] and unclear for one study [24].

### Quality assessment

Table 2 shows the quality assessments for the included studies. Risk of bias was high for 16 of 17 studies in the national population domain due to studies being conducted in only one province or region of a country, and so not being a close representation of the national population. Risk of bias was high for 12 of the 17 studies in the facility sampling domain due to sampled healthcare facilities not being a close representation of the province or region from which they were drawn. Risk of bias was low for 13 studies in the outcome measures domain, 14 studies in the visit sampling domain, 16 studies in the healthcare worker sampling domain and for all studies in the data collection and numerator and denominator domains.

**Table 2:** Quality assessment of included studies.

Study	Study population representative of national population	PHC facilities in study representative of PHC facilities in area	Visit sampling	Healthcare worker sampling	Data collection	Outcome measures	Numerator and denominator
Begum 2017	High	High	High	Unclear	Low	High	Low
Chueiri 2020	Low	Low	Low	Low	Low	Low	Low
Doyle 2019	High	High	Low	Low	Low	High	Low
Enato 2012	High	High	Low	Low	Low	Low	Low
Gupta 2014	High	High	High	Low	Low	Low	Low
Gupta 2015	High	High	High	Low	Low	Low	Low
Kamarudin 2012	High	High	Low	Low	Low	Low	Low
Kshirsagar 2019	High	High	Low	Low	Low	High	Low
Kumar 2018	High	High	Low	Low	Low	Low	Low
Mash 2012	High	Low	Low	Low	Low	Low	Low
Merali 2014	High	Low	Low	Low	Low	High	Low
Mohan 2014	High	High	Low	Low	Low	Low	Low
Olagundoye 2016	High	High	Low	Low	Low	Low	Low
Prabhune 2017	High	High	Low	Low	Low	Low	Low
Silva 2014	High	Low	Low	Low	Low	Low	Low
Swain 2017	High	Low	Low	Low	Low	Low	Low
Torres 2015	High	High	Low	Low	Low	Low	Low

PHC = primary health care; high = high risk of bias; unclear = unclear risk of bias; low = low risk of bias.

### Data pooling

The classification systems used were ICPC-2 for six studies [14, 22, 25, 27–29], ICD-10 for four studies (17–19, 24), in-house or unclear for six studies [13, 15, 16, 20, 21, 23] and Medical Dictionary for Regulatory Activities System Organ Class for one study [26]. Of the four studies which used ICD-10, three presented summed totals of the number of provider diagnoses in each chapter and only one included specific provider diagnoses. Unpooled results from studies which included specific provider diagnoses (one using ICD-10 [19] and three using in-house classification systems [16, 23, 24]), and from one study which included patient RFEs using an in-house classification system [29] are shown in S3 Appendix.

Four studies (Chueiri 2020 [14], Mash 2012 [22], Olagundoye 2016 [25] and Swain 2017 [28]) using ICPC-2 contained data on both RFEs and provider diagnoses in adults and were pooled. The top nine RFEs were ranked. The five most common RFEs were headache (N01), fever (A03), back symptom/low back symptom (L02, L03), cough (R05) and pain general/multiple sites (A01).

The top 10 provider diagnoses were ranked. The five most common provider diagnoses were hypertension, uncomplicated (K86), upper respiratory tract infection (R74),type 2 diabetes (T90), malaria (A73) and health maintenance/prevention (A98). [Table 3]. Raw data from the pooled studies are presented in S3 Appendix for reference.

**Table 3:** Top nine reasons for encounter and top 10 provider diagnoses in adults coded with ICPC-2, based on four studies.

Reasons for encounter	Rank score	Provider diagnoses*	Rank score
Headache (N01)	29	Hypertension, uncomplicated (K86)	37
Fever (A03)	27	Upper respiratory tract infection (R74)	23
Back symptom/low back symptom (L02, L03)	22	Type 2 diabetes (T90)	18
Cough (R05)	20	Malaria (A73)	10
Pain general/multiple sites (A01)	16	Health maintenance/prevention (A98)	10
Abdominal pain/cramps general (D01)	13	Allergic rhinitis (R97)	9
Vertigo/Dizziness (N17)	11	Pregnancy (W78)	9
Heart burn (D03)	9	HIV/AIDS (B90)	8
Leg/thigh symptom/complaint (L14)	8	Visual disturbance other (F05)	8
		Acute bronchitis/bronchiolitis (R78)	7
		Gastroenteritis/diarrhoea (D73, D11)	7
		Peptic ulcer (D86)	7

*Three provider diagnoses jointly had the tenth highest rank score so twelve provider diagnoses are presented here

## Discussion

Understanding patient RFEs and provider diagnoses in primary care in LMICs is of vital global health importance, as this information will guide the range of diagnostics required at the primary care level of each country. For example, malaria is often over-diagnosed in patients with febrile illness in settings in LMICs which lack access to appropriate diagnostics and presumptive treatment is given, leading to poor outcomes and the development of antimicrobial resistance [30–33]. Additionally, collecting such information could help to identify the diagnostic requirements of primary care healthcare providers in different settings and so guide the focus of future research and development in diagnostic technologies. In addition to guiding the choice of essential diagnostics, RFEs have a range of other uses. They can help to guide the choice of essential medicines, to understand care-seeking patterns in different settings, and they can be a valuable input for quality improvement efforts, as they are suggestive of the main competencies a health system needs to have to meet patient needs. The top five pooled RFEs in adults were headache, fever, back or low back symptoms, cough and pain general/multiple sites. The top five pooled provider diagnoses were uncomplicated hypertension, upper respiratory tract infection, type 2 diabetes, malaria and health maintenance/prevention. It is notable that the top 10 diagnoses varied between the studies substantially more than the top 10 RFEs. The diagnoses of HIV/AIDS and tuberculosis appeared only in Mash 2012 [22] from South Africa whereas malaria appeared only in Olagundoye 2016 [25] from Nigeria, in which it was the most common diagnosis. Pregnancy appeared only in Chueiri 2020 [14], though the prevalence of pregnancy in primary care depends on the demographics of the region or country and the organisation of maternal health services.

These findings show the importance of creating country-specific lists of essential diagnostics for primary care, as patients presenting with similar symptoms in different parts of the world may have substantially different disease patterns which require different diagnostic tools. Disease patterns may also vary substantially within countries, based on national disease burden. The development of multiplex or multi-disease molecular or point-of-care tests for fever-causing pathogens would be highly valuable but present a range of technical challenges [34].

A previous systematic review, mostly comprising data from HICs, had cough, back or spinal pain, unspecified abdominal condition, pharyngitis and dermatitis as the top five RFEs, with fever and headache only the sixth and seventh most common RFEs, respectively [9]. Upper respiratory tract infection and hypertension appeared in the top three provider diagnoses of the previous review, showing the overlap in diseases between LMICs and HICs, although the pathogens causing upper respiratory tract infections are likely to vary between locations.

Two of the three top provider diagnoses in this review are non-communicable diseases (NCDs), reflecting the increasing burden of NCDs in LMICs [35]. Uncomplicated hypertension causes no symptoms, as does type 2 diabetes in many cases, showing the importance of screening for diseases unrelated to patient RFEs. Diagnostics for hypertension and type 2 diabetes are simple and should be available at all PHCs, but diagnosis remains the weakest link in the cascade of care for these common NCDs in LMICs [36, 37].

The lack of psychological symptoms or disorders in the pooled lists of RFEs is striking. Despite mental and addictive disorders causing an estimated 7% of the entire global burden of disease [38], none of the top 9 pooled RFEs were psychological symptoms. Mash 2012 [22] reported the top 56 RFEs in their study, none of which were psychological symptoms, and concluded that “providers appears to be failing to recognise and treat mental health problems such as depression and anxiety disorders” in the four provinces they studied in South Africa. Swain 2017 [28] suggested that the lack of psychological RFEs among the 17 reported in their study may be due to stigma and culturally influenced health-seeking behaviour among the communities in their context in India. Chueiri 2020 [14] noted the unexpectedly low rate of psychological RFEs in their national survey of Brazil when compared to data from the Global Burden of Disease Study for Brazil [39] but did not suggest a reason for this finding.

The four studies which reported RFEs and provider diagnoses using ICPC-2 were at low risk of bias in most domains. However, three of the four were at high risk of bias in the national population domain, making comparison of their results problematic in a global perspective as they cannot be said to be representative even of their own national populations.

Where possible, future studies should aim to randomly sample primary care facilities from across their country of study, or to provide countrywide data from a national primary care database, if available. Ideally, studies should use ICPC-3 as it is designed for classifying primary care encounters, it allows reporting of both RFEs and specific diagnoses and unlike in-house classification systems, it is easily comparable between studies. ICPC-3 codes should be included with RFEs and specific diagnoses, with the numbers of included patients, visits, diagnoses and RFEs clearly identified. Data for each code should be presented separately, with no combining of codes. An electronic version of ICPC-3 is available for free on the ICPC-3 website [40]. If ICPC-3 cannot be used, another validated classification system such as ICD-11 should be used instead.

Strengths of this systemic review include a comprehensive literature search, detailed data on the characteristics of each study and an analysis of the strengths and limitations of different classification systems for recording reasons for primary care visits in LMICs. Additionally, using a non-parametric approach to pooling data, involving ranking RFEs and provider diagnoses and summing the ranks, enabled comparison of highly heterogenous data.

Limitations are that the variations in the classification systems used between studies made it difficult to directly compare most of the studies with each other. Only three studies could be pooled but even within these three, problems emerged with pooling. For example, in the RFEs Mash 2012 [23] provided combined data for back symptom (L02) and low back symptom (L03) whereas Olagundoye 2016 [18] and Swain 2017 [20] presented the two categories separately. Similarly, for provider diagnoses Mash 2012 combined the gastroenteritis presumed (D73) and diarrhoea (D11) categories whereas Olagundoye 2016 presented gastroenteritis presumed (D73) separately. It is unclear why Mash 2012 combined codes in a few select instances but presented most codes separately. Of the four pooled studies, one reported data from a three-month period so may not account for seasonal variation in disease presentation [25]. Additionally, we restricted our search to articles in English and French and did not include grey literature.

Given the substantial heterogeneity among the most common reasons for primary care visits both between and within countries, a country-by-country or subnational analysis may be preferable to a global review for informing country-specific EDLs. Additionally, national or subnational information may be stored in national health information systems, rather than published in peer-reviewed journals. However, quality of routinely collected data from health facilities is a concern, while research studies might offer more reliable data. Also, findings from this study show that such information is published in peer-reviewed journals, often with data from tens of thousands of patient encounters, and systematic reviews such as this are valuable for compiling and summarising the available data.

This systematic review found 17 studies from seven LMICs, none of them LICs, reporting data on RFEs and provider diagnoses in primary care. However, data from most studies could not be pooled. Headache, fever, back or low back symptoms, cough and pain general/multiple sites were the most common pooled RFEs but the diseases causing these symptoms varied substantially between settings, showing the importance of creating location-specific lists of essential diagnostics for primary care. Future studies should aim to sample primary care facilities from across their country of study and use ICPC-3 to report both patient RFEs and provider diagnoses. In particular, studies on the reasons for primary care visits in LICs are required.

## Supporting information

S1 Appendix Search strategies. (DOCX)

S2 Appendix Quality assessment tool. (DOCX)

S3 Appendix Unpooled results and raw data from pooled studies. (DOCX)

S4 Appendix PRISMA 2020 checklist. (DOCX)
