# Supplementary material for: Most common reasons for primary care visits in low- and middle-income countries: A systematic review
Source: PLOS Glob Public Health. 2022 May 2;2(5):e0000196. doi: 10.1371/journal.pgph.0000196 (PMC10022248; doi:10.1371/journal.pgph.0000196)
Supplement: S1 Appendix — (DOCX) [file pgph.0000196.s001.docx]

**S1 Appendix: Search strategies**

| Platform | Database(s) | Database coverage dates | # Results | Search Date |
| --- | --- | --- | --- | --- |
| OvidSP | Ovid MEDLINE ALL(R) | 1946 - | 5292  935 | 2019/12/11  Updated 2021/08/08 |
| OvidSP | EMBASE | 1996 - | 9719 | 2019/12/11 |
| OvidSP | Global Health | 1973 - | 3205 | 2019/12/11 |
| Web of Science | SCI-EXPANDED, SSCI, A&HCI, CPCI-S, CPCI-SSH, BKCI-S, BKCI-SSH, ESCI, CCR-EXPANDED, IC | 1900 - | 7300 | 2019/12/11 |
| EBSCOhost | CINAHL | 1937 - | 2649 | 2019/12/11 |
| VHL Regional Portal | LILACS | Inception - | 1290 | 2019/12/12 |
|  |  |  |  |  |
|  | **TOTAL NUMBER OF RECORDS** |  | 30390 |  |

**Number of records after duplicates removed: 22279 records**

Database: Ovid MEDLINE(R) ALL <1946 to December 10, 2019>

Search Strategy:

--------------------------------------------------------------------------------

1 Primary Health Care/ (74686)

2 Physicians, Primary Care/ (3191)

3 general practitioners/ or exp pediatricians/ or physicians, family/ or nurse practitioners/ or community health centers/ or ambulatory care information systems/ or ambulatory care/ or ambulatory care facilities/ or outpatient clinics, hospital/ or physician assistants/ or pediatric assistants/ (125034)

4 ((primary adj2 care) or community health*).ti,ab,kf,jw. or (primary health* or community care or community worker* or clinic or clinics or general practi* or family medicine or family practi* or family physician* or family doctor* or office visit* or medical office* or (ambulatory adj2 care) or pediatric? or paediatric? or internal medicine or personal physician* or Informal practi* or care provider* or prescriber? or midwife or midwives or (health adj2 worker*) or medical assistant* or health agent* or Outpatient care or county hospital* or (health* adj2 (center? or centre?)) or (community adj (center? or centre?)) or dispensary or dispensaries or pharmacy or pharmacies or nurse practitioner* or ((physician* or pediatric or paediatric) adj assistant*)).ti,ab,kf. (1050755)

5 1 or 2 or 3 or 4 (1120849)

6 "referral and consultation"/sn or health services accessibility/sn (20961)

7 ((reason* or ailments or causes or common or complaints or conditions or diagnoses or diseases or disorders or illnesses or motivations or problems or sicknesses or symptoms or syndromes) adj2 (attend* or encounter* or present* or visit* or consult*)).ti,ab,kf. (95793)

8 ((common or prevalent or frequent*) adj1 (ailments or diagnoses or complaints or conditions or diseases or disorders or illnesses or motivations or presentations or problems or reasons or sicknesses or symptoms or syndromes)).ti,kf. (1862)

9 ((disease* or illness* or morbidity) adj (burden or index or prevalence or rate? or pattern* or profile*)).ti,ab,kf. (38833)

Annotation: Added profile*

10 ((disease* or illness* or morbidity) adj2 (burden or index or prevalence or rate? or pattern* or profile*)).ti,kf. (9473)

Annotation: Added profile*

11 (episode* of illness or patient encounter record* or disease categor*).ti,ab,kf. (2913)

12 6 or 7 or 8 or 9 or 10 or 11 (163342)

13 (((primary adj2 care) or communit* or population) and (morbidity or health status)).ti,kf. (4324)

14 international classification of primary care.ti,ab,kf. (349)

15 (health services adj ("use" or usage or utili#ation)).ti,kf. (701)

16 12 or 13 or 14 or 15 (168099)

17 Developing Countries.sh. (73307)

18 (Africa or Asia or Caribbean or West Indies or South America or Latin America or Central America).hw,kf,ti,ab. (267717)

19 (Afghanistan or Albania or Algeria or Angola or Argentina or Armenia or Armenian or Azerbaijan or Bangladesh or Benin or Byelarus or Byelorussian or Belarus or Belorussian or Belorussia or Belize or Bhutan or Bolivia or Bosnia or Herzegovina or Hercegovina or Botswana or Brasil or Brazil or Bulgaria or Burkina Faso or Burkina Fasso or Upper Volta or Burundi or Urundi or Cambodia or Khmer Republic or Kampuchea or Cameroon or Cameroons or Cameron or Camerons or Cabo Verde or Cape Verde or Central African Republic or Chad or China or Colombia or Comoros or Comoro Islands or Comores or Mayotte or Congo or Zaire or Costa Rica or Cote d'Ivoire or Ivory Coast or Cuba or Djibouti or French Somaliland or "Afars and the Issas" or Dominica or Dominican Republic or East Timor or East Timur or Timor Leste or Ecuador or Egypt or United Arab Republic or El Salvador or Eritrea or eSwatini or Swaziland or Ethiopia or Fiji or Gabon or Gabonese Republic or Gambia or Gaza or Georgia Republic or Georgian Republic or Ghana or Gold Coast or Grenada or Guatemala or Guinea or Guiana or Guyana or Haiti or Honduras or India or Maldives or Indonesia or Iran or Iraq or Jamaica or Jordan or Kazakhstan or Kazakh or Kenya or Kiribati or Korea or Kosovo or Kyrgyzstan or Kirghizia or Kyrgyz Republic or Kirghiz or Kirgizstan or Lao PDR or Laos or Lebanon or Lesotho or Basutoland or Liberia or Libya or Macedonia or Madagascar or Malagasy Republic or Malaysia or Malaya or Malay or Sabah or Sarawak or Malawi or Nyasaland or Mali or Marshall Islands or Mauritania or Mauritius or Agalega Islands or Mexico or Micronesia or Middle East or Moldova or Moldovia or Moldovian or Mongolia or Montenegro or Morocco or Ifni or Mozambique or Myanmar or Myanma or Burma or Namibia or Nauru or Nepal or Nicaragua or Niger or Nigeria or Pakistan or Palestine or Paraguay or Peru or Philippines or Philipines or Phillipines or Phillippines or Romania or Rumania or Roumania or Russia or Russian or Rwanda or Ruanda or Saint Lucia or St Lucia or Saint Vincent or St Vincent or Grenadines or Samoa or Samoan Islands or Navigator Island or Navigator Islands or Sao Tome or Senegal or Serbia or Montenegro or Sierra Leone or Sri Lanka or Ceylon or Solomon Islands or Somalia or South Africa or Sudan or Suriname or Surinam or Syria or Tajikistan or Tadzhikistan or Tadjikistan or Tadzhik or Tanzania or Thailand or Togo or Togolese Republic or Tonga or Tunisia or Turkey or Turkmenistan or Turkmen or Tuvalu or Uganda or Ukraine or USSR or Soviet Union or Union of Soviet Socialist Republics or Uzbekistan or Uzbek or Vanuatu or New Hebrides or Venezuela or Vietnam or Viet Nam or West Bank or Yemen or Zambia or Zimbabwe or Rhodesia).hw,kf,ti,ab,cp,in,jw. (5583601)

20 ((developing or less* developed or under developed or underdeveloped or middle income or low* income or underserved or under served or deprived or poor*) adj (countr* or nation? or population? or world)).ti,ab,kf. (124615)

21 ((developing or less* developed or under developed or underdeveloped or middle income or low* income) adj (economy or economies)).ti,ab,kf. (516)

22 (low* adj (gdp or gnp or gross domestic or gross national)).ti,ab,kf. (236)

23 (low adj3 middle adj3 countr*).ti,ab,kf. (15222)

24 (lmic or lmics or third world or lami countr*).ti,ab,kf. (7239)

25 transitional countr*.ti,ab,kf. (158)

26 or/17-25 (5706264)

27 5 and 16 and 26 (7809)

28 limit 27 to yr="2009 -Current" (5292)

***************************
