# Supplementary material for: Most common reasons for primary care visits in low- and middle-income countries: A systematic review
Source: PLOS Glob Public Health. 2022 May 2;2(5):e0000196. doi: 10.1371/journal.pgph.0000196 (PMC10022248; doi:10.1371/journal.pgph.0000196)
Supplement: S2 Appendix — (DOCX) [file pgph.0000196.s002.docx]

**S2 Appendix: Quality assessment tool**

| **Quality assessment tool for cross-sectional studies**  Adapted from Hoy et Al [1]., NHLBI Quality Assessment Tool for Observational Cohort and Cross-sectional Studies [2], and Finley et Al. assessment checklist for their SR [3] | | |
| --- | --- | --- |
| Risk Of Bias Item | Criteria for answers | Additional notes and examples |
| **External validity** | | |
| 1. Was the study’s target population a close representation of the national population in relation to relevant variables, e.g. age, sex, occupation? | **Yes (LOW RISK)**: The study’s target population was a close representation of the national population.  **No (HIGH RISK)**: The study’s target population was clearly NOT representative of the  national population.  **Unclear or not reported** | LOW RISK:  1. Study was a national health survey of people 15 years and over and the sample was drawn from a list that included all individuals in the population aged 15 years and over.  HIGH RISK:  1. The study was conducted in one province only, and it is not clear if this was representative of the national population.  2. The study was undertaken in one village only and it is clear this was not representative of the national population. |
| 2. Were the healthcare facilities sampled a true or close representation of the target population? | **Yes (LOW RISK)**: The healthcare facilities sampled were a true or close representation of the target population.  **No (HIGH RISK)**: The healthcare facilities sampled were NOT a true or close representation of the target population.  **Unclear or not reported** | LOW RISK:  1. All PHCs in an area selected  2. A random sample of PHCs in an area using an acceptable randomization technique  HIGH RISK:  1. Not all PHCs in an area were included and their selection was not randomized  2. A convenience or purposive sample of PHCs was included |
| 3. Was some form of random selection used to select the sample of patient visits, OR, was a census undertaken? | **Yes (LOW RISK)**: A census  was undertaken, OR, some form of random selection was used to select the sample of patient visits.  **No (HIGH RISK)**: A census was NOT undertaken, AND some form of random selection was NOT used to select the sample.  **Unclear or not reported** | LOW RISK:  1. All patient visits in a selected period of time included  2. A random sample of patient visits in a selected period of time included using an acceptable randomization technique  HIGH RISK:  1. Not all patients were included and their selection was not randomized  2. A convenience sample of patients was included |
| 4. Was some form of random selection used to select the sample of healthcare workers, OR, was a census undertaken? | **Yes (LOW RISK)**: A census  was undertaken, OR, some form of random selection was used to select the professionals.  **No (HIGH RISK)**: A census was NOT undertaken, AND some form of random selection was NOT used to select the sample.  **Unclear or not reported** | LOW RISK:  1. All healthcare workers in the PHC were included  2. A random sample of healthcare workers in the PHC were included using an acceptable randomization technique  HIGH RISK:  1. Not all healthcare workers were included and their selection was not randomized  2. A convenience sample of healthcare workers was included |
| **Internal Validity** | | |
| **Data collection** | | |
| 5. Was the same mode of data collection used for all subjects? | **Yes (LOW RISK)**: The same  mode of data collection was used for all subjects.  **No (HIGH RISK)**: The same  mode of data collection was NOT used for all subjects.  **Unclear or not reported** | LOW RISK:  1. Data were collected from medical charts for everybody.  HIGH RISK:  1. Data were extracted from medical charts in the PHCCs, and from electronic medical records in the county hospitals. |
| **Assessment of the outcomes** | | |
| 6. Were the outcome measures clearly defined, valid, reliable, and implemented consistently across all study participants? | **Yes (LOW RISK)**: The study  instrument had been shown to have reliability and validity.  **No (HIGH RISK)**: The study  instrument had NOT been shown to have reliability or validity.  **Unclear or not reported** | LOW RISK:  1. The authors used a validated defined coding system (e.g. ICD-10, ICPC-2) to classify each condition across all participants.  2. The authors used a non-validated coding system, but their own system is defined, described and used for all participants.  HIGH RISK:  1. The authors used patient perspective complaints or diagnoses with no reference to a defined coding system.  2. The authors presented different coding for different groups without standardizing. NO |
| 7. Were the numerator(s) and denominator(s) for the parameter of interest appropriate? | **Yes (LOW RISK)**: The paper  presented appropriate  numerator(s) AND  denominator(s) for the parameters of interest.  **No (HIGH RISK)**: The paper  did present numerator(s) AND denominator(s) for the parameters of interest but one or more of these were inappropriate.  **Unclear or not reported** | LOW RISK:  1. There were no errors in the reporting of the numerator(s) AND denominator(s) for parameters of interest.  HIGH RISK:  1. In reporting the overall prevalence of low back pain (in both men and women), the authors accidentally used the population of  women as the denominator rather than the combined population. |
| [1] Hoy D, Brooks P, Woolf A, Blyth F, March L, Bain C, et al. Assessing risk of bias in prevalence studies: modification of an existing tool and evidence of interrater agreement. J Clin Epidemiol. 2012 Sep;65(9):934–9  [2] Study Quality Assessment Tools \| National Heart, Lung, and Blood Institute (NHLBI) [Internet]. [cited 2019 Oct 15]. Available from: https://www.nhlbi.nih.gov/health-topics/study-quality-assessment-tools  [3] Finley CR, Chan DS, Garrison S, Korownyk C, Kolber MR, Campbell S, et al. What are the most common conditions in primary care? Systematic review. Can Fam Physician Med Fam Can. 2018;64(11):832–40 | | |
