# Supplementary material for: Most common reasons for primary care visits in low- and middle-income countries: A systematic review
Source: PLOS Glob Public Health. 2022 May 2;2(5):e0000196. doi: 10.1371/journal.pgph.0000196 (PMC10022248; doi:10.1371/journal.pgph.0000196)
Supplement: S3 Appendix — (DOCX) [file pgph.0000196.s003.docx]

**S3 Appendix: Unpooled results** **and raw data from pooled studies**

*Unpooled results*

Enato 2012: top 10 provider diagnoses (in-house classification system)

| Provider diagnoses | Number (Percentage) |
| --- | --- |
| Malaria | 109 (21.3) |
| Typhoid | 59 (11.5) |
| Ulcer | 38 (7.4) |
| Trauma | 36 (7.0) |
| Hypertension | 33 (6.4) |
| Anaemia | 22 (4.3) |
| Respiratory tract infection | 21 (4.1) |
| Fever of unidentifiable cause | 18 (3.5) |
| Rheumatoid arthritis | 17 (3.3) |
| Gastroenteritis | 15 (2.9) |

Kamarudin 2012: top 10 provider diagnoses in all ages (ICD-10)

| Provider diagnoses | Percentage |
| --- | --- |
| Acute upper respiratory infection (EAR11) | 23.18 |
| Hypertension, w/o major complications (CAR14) | 12.70 |
| Preventive care (ADM06) | 7.19 |
| Type 2 diabetes, w/o complication (END06) | 7.04 |
| Disorders of lipoid metabolism (CAR11) | 4.53 |
| Dermatitis and eczema (SKN02) | 4.20 |
| Fever (GSI03) | 3.79 |
| Pregnancy and delivery, uncomplicated (FRE01) | 3.77 |
| Administrative concerns and non-specific laboratory abnormalities (ADM05) | 3.54 |
| Asthma, w/o status asthmaticus (ALL04) | 3.39 |

*Raw numbers could not be calculated due to unclear denominator

Merali 2014: top 5 provider diagnoses in adults (>15 years) (in-house classification system)

| Provider diagnoses | Number (Percentage) |
| --- | --- |
| Gastritis | 3565 (20.6) |
| Upper respiratory infection | 2582 (14.9) |
| Myalgia | 1433 (8.3) |
| Hypertension | 1199 (6.9) |
| Skin infection | 893 (5.2) |

Merali 2014: top 5 provider diagnoses in children (<15 years) (in-house classification system)

| Provider diagnoses | Number (Percentage) |
| --- | --- |
| Upper respiratory infection | 5707 (44.4) |
| Parasitism | 1712 (13.3) |
| Gastroenteritis | 1531 (11.9) |
| Skin infection | 951 (7.4) |
| Viral syndrome | 635 (4.9) |

Mohan 2014: top 10 provider diagnoses in all ages (in-house classification system)

| Provider diagnoses | Number (Percentage) |
| --- | --- |
| URI and tonsillitis | 6131 (15.59) |
| Pain abdomen | 5058 (12.86) |
| Skin infections (Infective and fungal) | 4443 (11.30) |
| Diarrhea | 3694 (9.39) |
| Nutritional deficiency diseases including Anemia | 2740 (6.97) |
| Bronchitis | 2262 (5.75) |
| Malaria | 1795 (4.56) |
| Eye diseases | 1638 (4.17) |
| Suppurative otitis media | 773 (1.97) |
| Urinary tract infection | 571 (1.45) |

Torres 2015: top 10 patient RFEs in adults (>18 years) (in-house classification system)

| Patient RFEs | Number (Percentage) |
| --- | --- |
| Pain | 163 (34.1) |
| Test evaluations/prescription renewal/medical certificates | 84 (17.6) |
| Hypertension | 25 (5.2) |
| Airway symptoms (coughing; sneezing/nasal congestion; shortness of breath/dyspnea; throat symptom) | 25 (5.2) |
| Skin complaints | 15 (3.1) |
| Anxiety/depression | 11 (2.3) |
| Vomiting/ diarrhea | 10 (2.0) |
| Palpable tumor | 10 (2.0) |
| Paresthesia | 9 (1.8) |
| Asthenia | 9 (1.8) |

*Raw data from pooled studies*

(i) RFEs

Chueiri 2020: top 9 reasons for encounter in adults (ICPC-2)

| Provider diagnoses | Number (Percentage) |
| --- | --- |
| Back symptoms/complaint (L02) | 263 (3.3) |
| Headache (N01) | 186 (2.3) |
| Leg/thigh symptom/complaint (L14) | 128 (1.6) |
| Throat symptoms/complaint (R21) | 104 (1.3) |
| Abdominal pain epigastric (D02) | 103 (1.3) |
| Abdominal pain/cramps general (D01) | 100 (1.2) |
| Pain general/multiple sites (A01) | 92 (1.1) |
| Vertigo/dizziness (N17) | 83 (1.0) |
| Cough (R05) | 69 (0.9) |

Mash 2012: top 9 reasons for encounter in adults (ICPC-2)

| Provider diagnoses | Number (Percentage) |
| --- | --- |
| Cough (R05) | 2821 (9.0) |
| Headache (N01) | 1500 (4.8) |
| Fever (A03) | 869 (2.8) |
| Sneezing/nasal complaint (R07, R08) | 624 (2.0) |
| Sore throat (R21) | 623 (2.0) |
| Back symptoms/low back symptom (L02. L03) | 589 (1.9) |
| Pain general/multiple sites (A01) | 585 (1.9) |
| Diarrhoea (D11) | 575 (1.8) |
| Abdominal pain/cramps general (D01) | 528 (1.7) |

Olagundoye 2016: top 9 reasons for encounter in adults (ICPC-2)

| Provider diagnoses | Number (Percentage) |
| --- | --- |
| Headache (N01) | 75 (18.7) |
| Fever (A03) | 69 (17.2) |
| Pain general/multiple sites (A01) | 52 (13.0) |
| Visual disturbance other (F05) | 47 (11.7) |
| Abdominal pain/cramps general (D01) | 37 (9.2) |
| Cough (R05) | 33 (8.2) |
| Lump/swelling localized (S04) | 23 (5.7) |
| Low back symptom/complaint (L03) | 21 (5.2) |
| Sensation disturbance other (N06) | 19 (4.7) |

Swain 2017: top 9 reasons for encounter in adults (ICPC-2)

| Provider diagnoses | Number (Percentage) |
| --- | --- |
| Fever (A03) | 279 (11.4) |
| Heart burn (D03) | 198 (8.1) |
| Vertigo/Dizziness (N17) | 88 (3.6) |
| Muscle pain (L18) | 64 (2.6) |
| Low back symptom/complaint (L03) | 54 (2.2) |
| Weakness/tiredness general (A04) | 51 (2.1) |
| Diarrhea (D11) | 49 (2.0) |
| Cough (R05) | 37 (1.5) |
| Teeth gum symptom (D19) | 32 (1.3) |

(ii) Provider diagnoses

Chueiri 2020: top 10 provider diagnoses in adults (ICPC-2)

| Provider diagnoses | Number (Percentage) |
| --- | --- |
| Health maintenance/prevention (A98) | 583 (7.2) |
| Pregnancy (W78) | 471 (5.9) |
| Hypertension uncomplicated (K86) | 317 (3.9) |
| Diabetes non-insulin dependent (T90) | 151 (1.9) |
| Influenza (R80) | 103 (1.3) |
| Allergy/allergic reactions NOS (A92) | 82 (1.0) |
| Cystitis/urinary infection other (U71) | 48 (0.6) |
| Lipid disorder (T93) | 43 (0.5) |
| Depressive disorder (P76) | 33 (0.4) |
| General disease NOS (A99) | 30 (0.4) |

Mash 2012: top 10 provider diagnoses in adults (ICPC-2)

| Provider diagnoses | Number (Percentage) |
| --- | --- |
| Hypertension, uncomplicated (K86) | 2957 (12.0) |
| Upper respiratory tract infection (R74) | 1306 (5.3) |
| HIV/AIDS (B90) | 961 (3.9) |
| Type 2 diabetes (T90) | 946 (3.9) |
| TB (A70) | 862 (3.6) |
| Osteoarthritis (L91) | 681 (2.8) |
| Gastroenteritis/diarrhoea (D73, D11) | 530 (2.2) |
| Asthma (R96) | 491 (2.0) |
| Acute tonsillitis (R76) | 485 (2.0) |
| Epilepsy (N88) | 454 (1.9) |

Olagundoye 2016: top 10 provider diagnoses in adults (ICPC-2)

| Provider diagnoses | Number (Percentage) |
| --- | --- |
| Malaria (A73) | 72 (18.0) |
| Hypertension, uncomplicated (K86) | 47 (11.7) |
| Visual disturbance, other (F05) | 22 (5.5) |
| Peptic ulcer (D86) | 14 (3.5) |
| Upper respiratory tract infection (R74) | 13 (3.2) |
| Hypertension, complicated (K87) | 11 (2.7) |
| Cystitis (U71) | 11 (2.7) |
| General diseases not elsewhere spec'd (A99) | 11 (2.7) |
| Conjunctivitis, infectious (F70) | 9 (2.2) |
| Refractive error (F91) | 9 (2.2) |

Swain 2017: top 10 provider diagnoses in adults (ICPC-2)

| Provider diagnoses | Number (Percentage) |
| --- | --- |
| Hypertension, uncomplicated (K86) | 206 (10.2) |
| Allergic rhinitis (R97) | 152 (7.5) |
| Upper respiratory tract infection (R74) | 101 (5.0) |
| Acute Bronchitis/Bronchiolitis (R78) | 91 (4.5) |
| Viral (A77) | 77 (3.8) |
| Gastrointestinal Infection (D70) | 40 (2.0) |
| Diabetes non‑insulin Dependant (T90) | 34 (1.7) |
| Gastro enteritis presumed (D73) | 28 (1.4) |
| Skin disease other (S99) | 22 (1.1) |
| Cystitis/Urinary infection other (U71) | 22 (1.1) |
